# Evaluation of dosimetric impact and plan robustness of MLC motion and setup errors in SRS for multiple brain metastases

**DOI:** 10.1002/acm2.70697

**Published:** 2026-07-29

**Authors:** Huijun Xu, Hong Zhang, Weiguang Yao, Jinghao Zhou, Yannick Poirier, Junliang Xu, Stewart Becker, Guozhen Luo, Mark Mishra, Shifeng Chen, Baoshe Zhang

**Affiliations:** ^1^ Department of Radiation Oncology University of Maryland School of Medicine Baltimore Maryland USA; ^2^ Department of Radiation Oncology Vanderbilt University Medical Center Nashville Tennessee USA

**Keywords:** margin, multiple brain metastases, robustness, SRS

## Abstract

**Background:**

HyperArc is an effective technique for treating multiple small brain metastases using high‐definition, precise radiation. However, the small size of individual lesions makes treatment vulnerable to inherent inaccuracies from beam‐limiting devices such as multi‐leaf collimators (MLCs), as well as patient setup errors. These uncertainties must be thoroughly evaluated prior to administering a single‐fraction, high‐dose HyperArc treatment to ensure optimal clinical outcomes.

**Purpose:**

To comprehensively evaluate the robustness of HyperArc VMAT plans for multiple brain metastases (MBT) against (i) high‐definition MLC leaf positional errors and (ii) patient rotational and translational setup variations.

**Methods and materials:**

A retrospective analysis of 34 MBT plans from 30 patients with 123 lesions treated using HyperArc VMAT plans was performed. Prescription doses (21–24 Gy) targeted PTVs (GTV + 1 mm), with each plan addressing 2–7 brain metastases (< 2 cm). D_100%_ for GTV and V_12Gy_ for normal brain were re‐evaluated under simulated (i) 20 MLC positional errors (0.2–1 mm) and (ii) 26 patient rotational (Yaw/Pitch/Roll by 1°) and 26 translational errors (SI/LR/AP by 1 mm). The impact of target distance to isocenter (DTI), target volume, and plan modulation factor on plan robustness was assessed. Replans with larger PTV margins, 1.5 and 2 mm, were investigated for a proper PTV margin for the worst‐case scenarios.

**Results:**

Per our cohort, D_100%_ of GTV is highly sensitive to both MLC and patient errors. Their dosimetric effects follow linear regressions: 1 mm shift reduces GTV D_100%_ by 8%; 1 mm symmetric MLC openings increase GTV D_100%_ by > 22%, while 1 mm symmetric closings decrease it by > 25.8% per 1 mm; Patient rotation/translation impacts GTV D_100%_ by 0.04–1.28% per 1° or ∼7% per 1 mm. Normal brain V_12Gy_ is only sensitive to MLC errors, increasing slightly with shifts and significantly with symmetric openings (+5cc/1 mm), but decreasing with closings (−2.77cc/1 mm). DTI shows minimal correlation with MLC shifts but is affected by patient setup uncertainties. Large targets amplify dose sensitivity to symmetric MLC errors, whereas plan modulation factor shows no significant impact. A minimum 1 mm margin is required to absorb these uncertainties, with 2 mm margins recommended in the worst cases to ensure treatment accuracy.

**Conclusions:**

Overall, the target dose and V_12Gy_ for HyperArc VMAT plans are more significantly affected by symmetric MLC opening and closing errors than by patient rotational setup errors. The pronounced effects of these errors highlight the importance of rigorous plan robustness verification, accounting for target size, margins, and DTI.

## 1 INTRODUCTION

Stereotactic radiosurgery (SRS) uses sophisticated, three‐dimensional computerized imaging to precisely deliver photon beams with a highly concentrated dose of radiation to a precise target in a single session.^1^ In recent decades, stereotactic radiosurgery/radiotherapy (SRS/SRT) has demonstrated durable tumor control and symptomatic relief with acceptable toxicity in patients with multiple brain lesions. Notable platforms for delivering SRS include the Gamma Knife and the linear accelerator (linac)‐based system.

One recent technological advancement in linac‐based SRS is HyperArc VMAT module from Varian Medical Systems (Varian Medical Systems, Inc, Palo Alto, CA). This module capitalizes on all available degrees of freedom to orchestrate dynamic gantry and couch movements, optimizing non‐coplanar beam utilization. Hyperarc allows a fully automated integration of treatment delivery technology with the Eclipse treatment planning system. Planning of stereotactic treatment for brain is optimized via highly conformal dose distributions, rapid dose fall‐off, excellent sparing of adjacent critical organs, and highly accurate treatments.^2^, ^3^, ^4^HyperArc has exhibited some advantages over the conventional planning methodologies used in for example, gamma knife^5^ and proton.^6^ Compared to regular VMAT, HyperArc yields superior conformity and OAR sparing,^3^, ^5^, ^7^, ^8^, ^9^ or maintains similar plan quality while significantly accelerating the optimization process.^10^, ^11^ Consequently, HyperArc has been proven particularly advantageous for less experienced planners.^12^

However, unlocking the full potential of HyperArc requires careful consideration of several key factors. First, the plan quality of HyperArc depends on the MLC width, as the narrower MLC results in higher conformity, steeper dose gradient, and improved normal tissue sparing.^13^ Also, MLC tends to have more aperture effect than a cone for small SRS target plans.^14^ The choice of collimator angle contributes to reduced brain tissue doses and heightened treatment efficacy.^15^ Furthermore, there are some potential uncertainties/errors associated with HyperArc. Prentou *et al* ^16^ simulated systematic MLC positioning offsets, with symmetrical opening (closing) of both leaf banks by 0.09 up to 0.94 mm; They found plan quality of HyperArc is very sensitive to sub‐millimeter leaf positional inaccuracies, and this sensitivity increases considerably compared to the similar studies for IMRT/VMAT.^17^, ^18^, ^19^, ^20^ Some studies^21^, ^22^have pointed out that patient rotation usually results in a loss of target coverage, particularly for smaller targets and isocenter placement farther away from the lesion. While some studies^23^, ^24^ found that rotational setup errors for multiple brain metastases caused PTV underdosage, and significant increases of V_10Gy_ and V_16Gy_. To mitigate such dosimetric downgrade in target coverage, these researchers suggest using a six degree of freedom (6DoF) couch for set‐up and surface tracking during beam delivery,^21^ or introducing additional isocenter(s) to limiting the lesion‐to‐isocenter distance to ≤4 cm.^22^ Besides, Intra‐fractional motion error during HyperArc SRS on patients with brain metastases is also an important factor to consider.^25^, ^26^

Although the impact of uncertainties on HyperArc has been investigated by the above researchers, comprehensive validation of SRS HyperArc treatment robustness against MLC and patient rotational setup uncertainties remains limited. Furthermore, the significance of these uncertainties may depend on tumor size, location, and plan characteristics on these uncertainties, but these have rarely been examined in detail. This paper scrutinizes how target distance from the isocenter, lesion size, margin, and target modulation influence susceptibility to MLC errors and patient rotational uncertainties through statistical analysis. This study also provides a guideline of margin selection to absorb uncertainties aroused by MLC and rotations.

## 2 MATERIALS AND METHODS

### 2.1 Patient selection

In this IRB‐approved study, we retrospectively analyzed data from 30 randomly‐selected SRS patients with 34 HyperArc plans. There were 2–7 brain metastases lesions treated for each patient, yielding a total of 123 lesions to be analyzed in this study. Each patient received a single‐fraction treatment of 18 to 24 Gy to the PTV using Linac‐based SRS with high‐definition (HD) MLC. Patients were simulated in the supine position using the indexed Q‐Fix SRS Encompass mask system (CQ Medical, PA, USA), with 1.0 mm slice thickness CT scans acquired from the vertex to the shoulders. For target delineation, a contrast‐enhanced 3D T1‐weighted MRI (1.0 mm slice thickness) was acquired within one week of simulation and rigidly co‐registered to the planning CT; the accuracy of this fusion was manually reviewed and approved by the treating physician prior to contouring GTV and normal brain tissue (defined as the brain minus GTV). PTV is an isotropic expansion from GTV by 1 mm margin.

Treatment planning was conducted in the Eclipse HyperArc system (Eclipse version 16.1). The planning goal was to deliver the prescription dose (Rx) to 100% of the GTV. Their PTVs were planned to achieve 99% coverage. Dose precision and fall‐off were evaluated using Conformity index (CI)^27^ and Gradient Index (GI),^28^ with target values of < 1.25 and < 4.0, respectively. Dose limits for critical organs included 10 Gy to 0.5 cc for the brainstem, 8 Gy for the optic chiasm, and < 10 cc of normal brain tissue receiving ≥12 Gy. Some patients had received prior treatment more than a year before this SRS treatment, while others had no previous treatment history.

**FIGURE 1:**
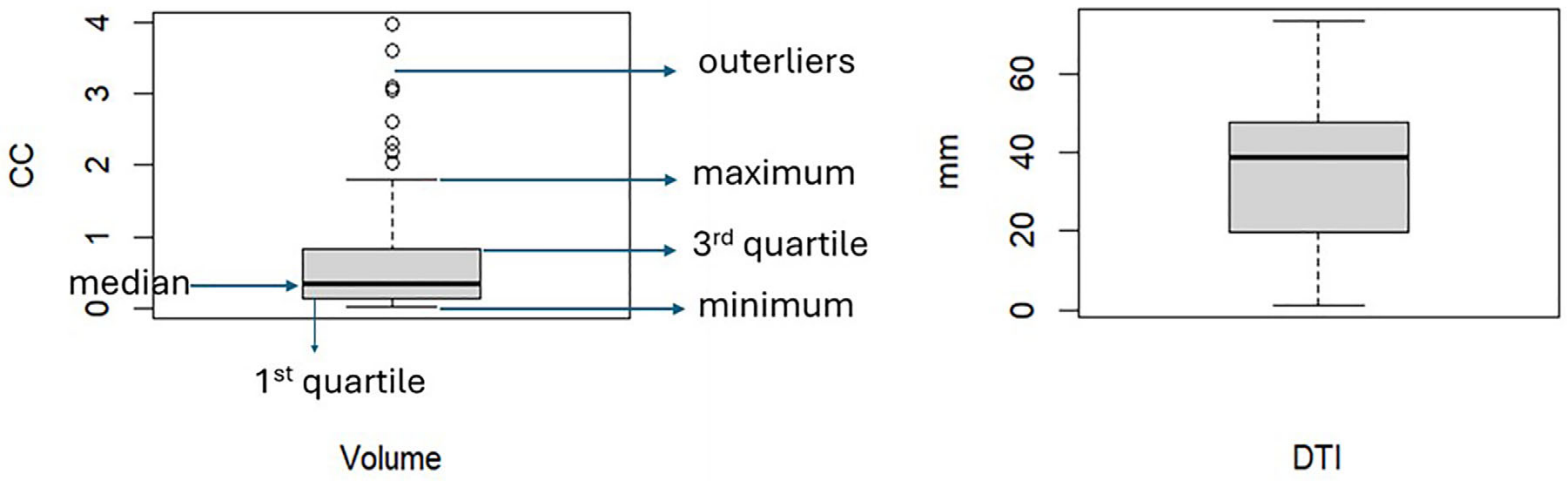
The left diagram shows the volume (cc) distribution of all 123 GTVs in the study; the right diagram shows the DTI (mm) distribution of those GTVs. 1^st^ quartile and 3^rd^ quartile refer to 25% and 75% percentile, respectively.

The distributions of all the lesion volumes and their locations (distance to isocenter (DTI)) are illustrated in Figure 1.

### 2.2 Simulation of MLC errors and rotational uncertainties

Starting with the original patient plans exported as RT‐Plan DICOM files from the ARIA Oncology Information System (Varian, Palo Alto, California), we used an in‐house program to adjust the positions of each pair of MLC leaves based on predefined errors. When the gap between any pair of leaves was smaller than the system's minimum threshold (e.g., 0.5 mm for our treatment planning system), we set the leaf gap to this minimum. Leaf positions of any static leaves during treatment were maintained as‐is, often remaining outside the treatment field.

Four MLC error types were analyzed: unidirectional shifts (left‐to‐right or right‐to‐left), modeling a leaf‐carriage calibration offset, and symmetric opening or closing, modeling a DLG miscalibration or uniform leaf‐end offset affecting both banks. Five error magnitudes — 0.2, 0.4, 0.6, 0.8, and 1.0 mm — were tested for each type, yielding 20 combinations that span from routinely observed sub‐millimeter offsets to the vendor‐specified HD‐120 MLC leaf‐position accuracy limit of ± 1.0 mm (reproducibility ± 0.5 mm).^29^ To characterize the effect of quasi‐random MLC positional uncertainty, we also simulated 100 HyperArc plans with MLC leaf position errors drawn from a uniform random distribution over the range of 0–1.0 mm. The modified RT‐Plan DICOM files were re‐imported into Eclipse for dose recalculation on an Edge linear accelerator equipped with HD‐120 MLCs.

Additionally, we simulated patient rotational and translational setup errors and the rotational errors in three directions (yaw, pitch, and roll) centered around the beam isocenter. Using specified values for each rotational axis, a program generated a rigid registration DICOM file. This registration was applied to replicate the original plan onto a new CT set, identical to the initial CT set, for consistent comparison. Dose calculations were then performed on these new plans for analysis. The typical residual errors after 6DOF correction in rotational and translational setups are within 1 degree and 1 mm, respectively.^30^, ^31^ In our clinical practice, SRS patient positioning is performed by registering the onboard cone‐beam CT (CBCT) with the planning CT. During treatment delivery, patient motion is continuously monitored using the Vision RT surface guidance system (Vision RT, London, England). If translational motion exceeds 1 mm or rotational motion exceeds 1° relative to the CBCT‐based setup position, treatment is paused, and the patient is repositioned with a newly acquired CBCT scan. Accordingly, in this study, rotational setup errors were modeled with a magnitude of 1° along each rotational axis, resulting in 26 possible combinations of rotations across the three axes. For instance, a combination of [−1, 1, 1] signifies a yaw error of −1 degree, pitch error of 1 degree, and roll error of 1 degree. Similarly, 26 combinations of translational errors with 1 mm offset in 1,2 and 3 DOF scenarios were simulated here. Table 1 lists the categories of our simulation data based on their uncertainties.

**TABLE 1:** Nine categories of our simulation data: C1:MLC shift (left and right shift with magnitudes of 0.2 mm, 0.4 mm, 0.6 mm, 0.8 mm, and 1.0 mm), C2: MLC symmetrical closing (magnitude of 0.2 mm, 0.4 mm, 0.6 mm, 0.8 mm, and 1.0 mm), C3: MLC symmetrical opening (magnitude of 0.2 mm, 0.4 mm, 0.6 mm, 0.8 mm, and 1 mm), C4–C6: Rotations of 1 degree with 1, 2, and 3 degrees of freedom and C7–C9 translation of 1 mm with 1, 2 and 3 degrees of freedom.

Category	Error Type	Description	Magnitude
C1	MLC Shift	Left / right (systematic)	0.2, 0.4, 0.6, 0.8, 1.0 mm
C2	MLC Movement	Symmetrical closing	0.2, 0.4, 0.6, 0.8, 1.0 mm
C3	MLC Movement	Symmetrical opening	0.2, 0.4, 0.6, 0.8, 1.0 mm
C4	Rotation	1 degree of freedom	1°
C5	Rotation	2 degrees of freedom	1° per DOF
C6	Rotation	3 degrees of freedom	1° per DOF
C7	Translation	1 degree of freedom	1 mm
C8	Translation	2 degrees of freedom	1 mm per DOF
C9	Translation	3 degrees of freedom	1 mm per DOF

### 2.3 Plan Modulation

Like other VMAT plans, HyperArc plans use the synchronized motion of multi‐leaf collimator leaves to generate desired dose distributions for tumor targets with complex shapes, in particular, for multiple spatially separated tumor targets. To analyze how plan modulation might affect plan quality variation induced by MLC positional errors and patient rotational/translational errors, we define a modulation factor as below. For each control point with certain gantry angle/collimator angle/couch rotation, a conformal aperture, S_cp_, is the MLC shape conforming to the BEV of each individual tumor target. The useful MLC aperture of each control point is defined as the intersection of the conformal aperture and the plan MLC aperture, A_cp_, at each control point, i,e, S_cp_ ∩ A_cp_. To quantify the degree of plan modulation, we introduced the following expression (Equation 1):

(1) $$M=\sum_{beam=1}^{n}\frac{MU_{beam}}{MU_{plan}}\frac{\sum_{cp=1}^{m}MU_{cp}\left(S_{cp}\cap A_{cp}\right)}{\sum_{cp=1}^{m}MU_{cp}S_{cp}}$$

where, MU_cp_ is the MU value for a corresponding control point, MU_beam_ is the sum of all the control points of a beam, MU_plan_ is the sum of all the beams of a plan. This definition of plan modulation factor is a MU‐weighted measure of MLC aperture's openness relative to the tumor target. For a conformal VMAT, M is very close to 1; for a highly modulated VMAT, M is far less than 1.

### 2.4 Margin selection

HyperArc‐based VMAT workflows have been widely reported in the literature. In these studies,^32^, ^33^ GTV‐to‐PTV margins ranging from 0 to 2 mm have been used, with 1 mm being the most commonly reported margin in clinical HyperArc cohorts. Here for most patients, a 1.0 mm PTV margin was applied to the GTV for treatment planning. To assess worst‐case scenarios in which this margin may not adequately account for uncertainties, additional plans were created using expanded margins of 1.5 mm and 2 mm. Target coverage and OAR doses were then recalculated for these larger‐margin scenarios. It should be noted that when a 1.5 mm margin is applied in Eclipse, the margin in the superior–inferior direction is limited to 1 mm due to the CT slice thickness (1 mm).

### 2.5 Multiple linear regression modelling

For each GTV, the following parameters were extracted from treatment planning systems:

- **GTV** D_100%_ **Change (%)**: Difference in D_100%_ between baseline and plans with deviations. (D_100%_)
- **GTV Volume (CC)**: Volume of the gross tumor. (Vol)
- **Distance to Isocenter (cm)**: Euclidean distance from the GTV centroid to the beam isocenter. (DTI)

Throughout this study, a ‘group’ denotes a single discrete simulated error scenario — that is, one specific combination of error type, affected axis/direction, and magnitude — and each group is nested within one of the nine categories (C1–C9) defined in Table 1. The MLC error categories (C1–C3) comprise 20 groups (4 error types × 5 magnitudes), while the rotational (C4–C6) and translational (C7–C9) categories each comprise 26 groups (6 one‐DOF, 12 two‐DOF, and 8 three‐DOF combinations). For each group, the multiple linear regression model (Equation 2) was fit across all 123 lesions. The model is defined as:

(2) D100%=β0+β1·Vol+β2·DTI

Where:

- D_100%_: predicted change in dose coverage of the GTV
- β_0_: **Intercept** — the predicted D_100%_ change when both predictors are zero
- β_1_: **Volume coefficient—**estimated change in D_100%_ per unit increase in GTV volume (CC)
- β_2_: **Distance coefficient** — estimated change in D_100%_ per unit increase in distance to isocenter (mm)

Model fitting and statistical analysis were performed in R (version 4.5.1) using the lm() function.

The β_0_, β_1_ and β_2_ for each group were calculated, and the β_0_, β_1_ and β_2_ with *p* value less than 0.05 were compared.

## 3 RESULTS

### 3.1 Dosimetric effect on GTV

**FIGURE 2:**
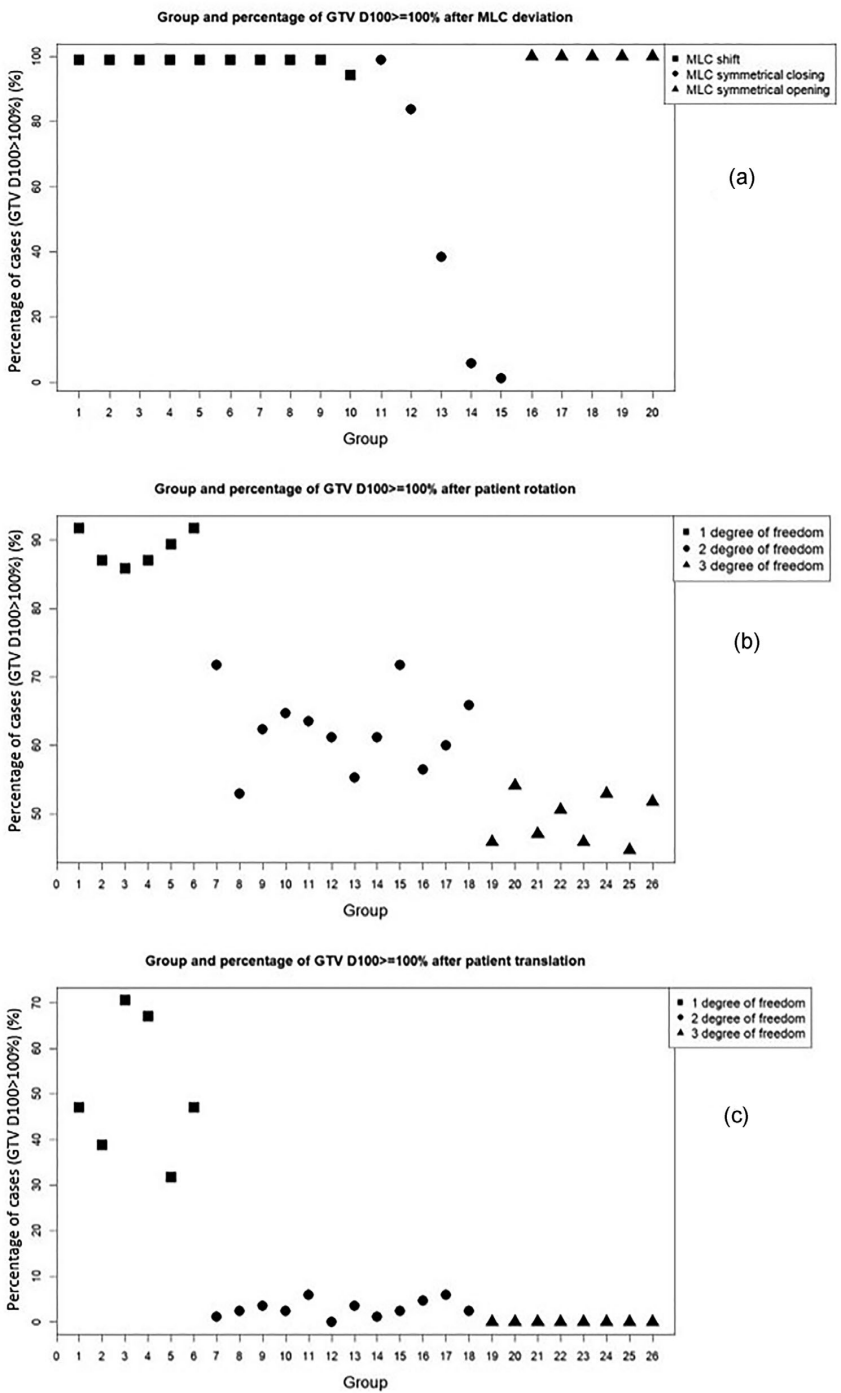
Percentage of cases in which D_100%_ of GTV is greater than or equal to the prescribed dose following a) simulated MLC errors and b) simulated patient rotational errors and c) simulated patient translational errors. The MLC symmetrical closing shows the most significant dosimetric effect on GTV D_100%_. GTV D_100%_ is degraded with higher degree of freedom rotation of rotational errors. GTV D_100%_ is dramatically lowered with 2DOF or 3DOF translation errors. MLC groups 1–20 span C1 single‐sided shifts (Groups 1–10; left/right pairs at 0.2–1.0 mm), C2 symmetric closings (Groups 11–15), and C3 symmetric openings (Groups 16–20), each at 0.2–1.0 mm (see Table 4). Rotation groups (26 total) apply a 1° error in 1‐, 2‐, and 3‐DOF scenarios (6, 12, and 8 combinations); translation groups (26 total) apply a 1 mm offset in 1‐, 2‐, and 3‐DOF scenarios (6, 12, and 8 combinations). These are listed in Supplementary Tables S1 and S2, respectively.

Our data show that GTV D_100%_ is highly sensitive to some MLC positional errors and patient setup uncertainties, among all factors, MLC symmetrical closing shows the most significant dosimetric effect on GTV D_100%_ (Figure 2a): The GTV D_100%_ coverage is downgraded significantly, while GTV D_100%_ is quite stable under other MLC positional errors. With rotational errors, GTV D_100%_ continues to drop as the errors DOF get higher (Figure 2b), and is dramatically reduced to nearly 0 with 2DOF or 3DOF translation errors (Figure 2c).

Please note that GTV D_100__%_ was the primary target metric in this study, simulating various uncertainties, as it represents the clinical planning objective of HyperArc SRS at our institution. GTV D_98%_ and D_95%_ only served as secondary endpoints as they are less sensitive to small geometric perturbations and peripheral underdosage. The variation in D95% and D98% was consistently smaller than the variation in D_100%_, and the gap depended on the type of uncertainty. For MLC uncertainty, the two metrics tracked D_100%_ closely (D_95%_ variation ≈ 89.6% and D_98%_ ≈ 92.5% of the D_100%_ variation). For translational uncertainty, both were far less sensitive (D_95%_ ≈ 54.6% and D_98%_ ≈ 66.3%), and rotational uncertainty showed the smallest variations relative to D_100%_ (D_95%_ ≈ 45.1% and D_98%_ ≈ 60.9%).

We also find that the dosimetric effects of the simulated errors follow linear regressions. Several error categories showed statistically significant coefficients. Based on equation 2, specifically, categories C1, C2, C3, C7, C8, and C9 demonstrated significant intercepts (β_0_) with *p*‐values < 0.05, indicating a strong baseline impact; Volume‐related coefficients (β_1_) were significant for categories C2 and C3 (*p* < 0.05), while rotational degree‐of‐freedom coefficients (β_2_) were significant for categories C4, C5, and C6 (*p* < 0.05).

The linear regressions provide a straightforward estimate of the dosimetric effect on GTV D_100%_. As table 2 shows, a 1 mm MLC shift error (C1) reduced D_100%_ by 8.6% (R^2^ = 0.91), while symmetric closings (C2) decreased it by 25.3%/mm and openings (C3) increased it by 23.3%/mm (both R^2^ = 1). These results highlight the critical dosimetric impact of small MLC positional deviations. The effect of rotation setup errors (C4–C6) did not show a significant correlation with GTV D_100%._ Translational errors showed strong effects: D_100%_ declined 9.0%, 16.6%, and 23.7% for 1–3 degrees of freedom (C7–C9), averaging a 7.3% reduction per added degree (R^2^ = 1), indicating a cumulative effect of increased setup complexity.

**TABLE 2:** Dosimetric effects of MLC errors and patient setup errors on GTV D_100%_, with R^2^ values reported for significant linear relationships.

Error Type	Category	Error Description	Change in GTV D_100%_	R^2^	*p*‐value
MLC Errors	C1	Single‐sided MLC shift	−8.6% /1 mm	0.91	< 0.001
	C2	Symmetric MLC closing	−25.3% /1 mm	1.00	< 0.001
	C3	Symmetric MLC opening	+23.3% /1 mm	1.00	< 0.001
Translational Setup Errors	C7	1 DOF	−9.0% /1 mm	–	Average < 0.001
	C8	2 DOF	−16.6% /1 mm	–	Average < 0.001
	C9	3 DOF	−23.7% /1 mm	–	Average < 0.001
	–	Incremental effect	−7.3% per DOF	1.00	0.013

**FIGURE 3:**
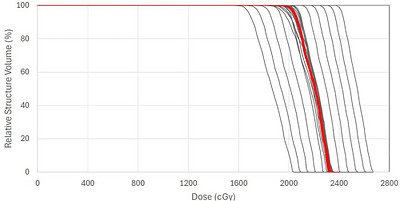
The grey lines represent DVH curves for a PTV target across 20 treatment plans incorporating systematic MLC positional shifts (± 0.2 mm, ± 0.4 mm, ± 0.6 mm, ± 0.8 mm, ± 1.0 mm), including the original HyperArc plan. The red lines represent DVH curves from 100 plans with randomly distributed MLC leaf position errors ranging from 0 mm to 1 mm uniformly.

In Figure 3, the simulated randomly distributed MLC positional errors for 100 HyperArc plans did not show significant statistical variability as what is shown in Figure 2c. It demonstrates that realistically distributed sub‐millimeter errors produce dosimetric variability that is bounded by, and smaller than, that of the systematic fixed‐error scenarios.

Beyond MLC and setup errors, DTI and tumor volume also affect HyperArc robustness (Table 3). For each 1 mm increase in DTI, D_100%_ decreased by 0.1%, 0.2%, and 0.3% in C4–C6, respectively. With DTI fixed at 1 mm, each added rotational degree reduced D_100%_ by 0.09% (R^2^ = 1). In this cohort, DTI ranged up to 73 mm (Figure 1), limiting the maximum rotational effect to ∼0.3% per mm under 3‐DOF rotation; larger DTI values would yield proportionally greater impact. Tumor size further modulated MLC sensitivity: a 1 cc volume increase raised D_100%_ by 5.5% for 1 mm closings (C2) but lowered it by 5.9% for 1 mm openings (C3, both R^2^ = 1). Smaller tumors thus show greater resistance to symmetric MLC deviations, highlighting the need for anatomy‐specific robustness evaluation.

**TABLE 3:** Dependence of GTV D_100%_ on DTI and tumor volume, with R^2^ values reported for significant linear relationships.

Factor	Category	Description	Change in GTV D_100%_	R^2^	*p*‐value
DTI	C4	Rotational setup error (1 DOF)	−0.1% / +1.0 mm DTI	–	Average 0.0037
	C5	Rotational setup error (2 DOF)	−0.2% / +1.0 mm DTI	–	Average < 0.001
	C6	Rotational setup error (3 DOF)	−0.3% / +1.0 mm DTI	–	Average < 0.001
	–	Rotational DOF effect	−0.09% / +1 DOF (with DTI = 1 mm)	1.00	0.025
Tumor Volume	C2	Symmetric MLC closing	+5.5% / +1.0 cc volume (with 1.0 mm closing)	1.00	< 0.001
	C3	Symmetric MLC opening	−5.9% / +1.0 cc volume (with 1.0 mm opening)	1.00	< 0.001

Our study did not find either positive or negative correlations between our defined plan modulation factor and dosimetric metrics. No correlation is observed for the plan modulation factor with either target volume or DTI metrics. For all these HyperArc plans, the plan modulation factor varies from 0.31 to 0.95. That is, plan quality variations from our simulated errors are relatively independent of plan modulation.

### 3.2 Dosimetric effect on normal brain V_12Gy_

**FIGURE 4:**
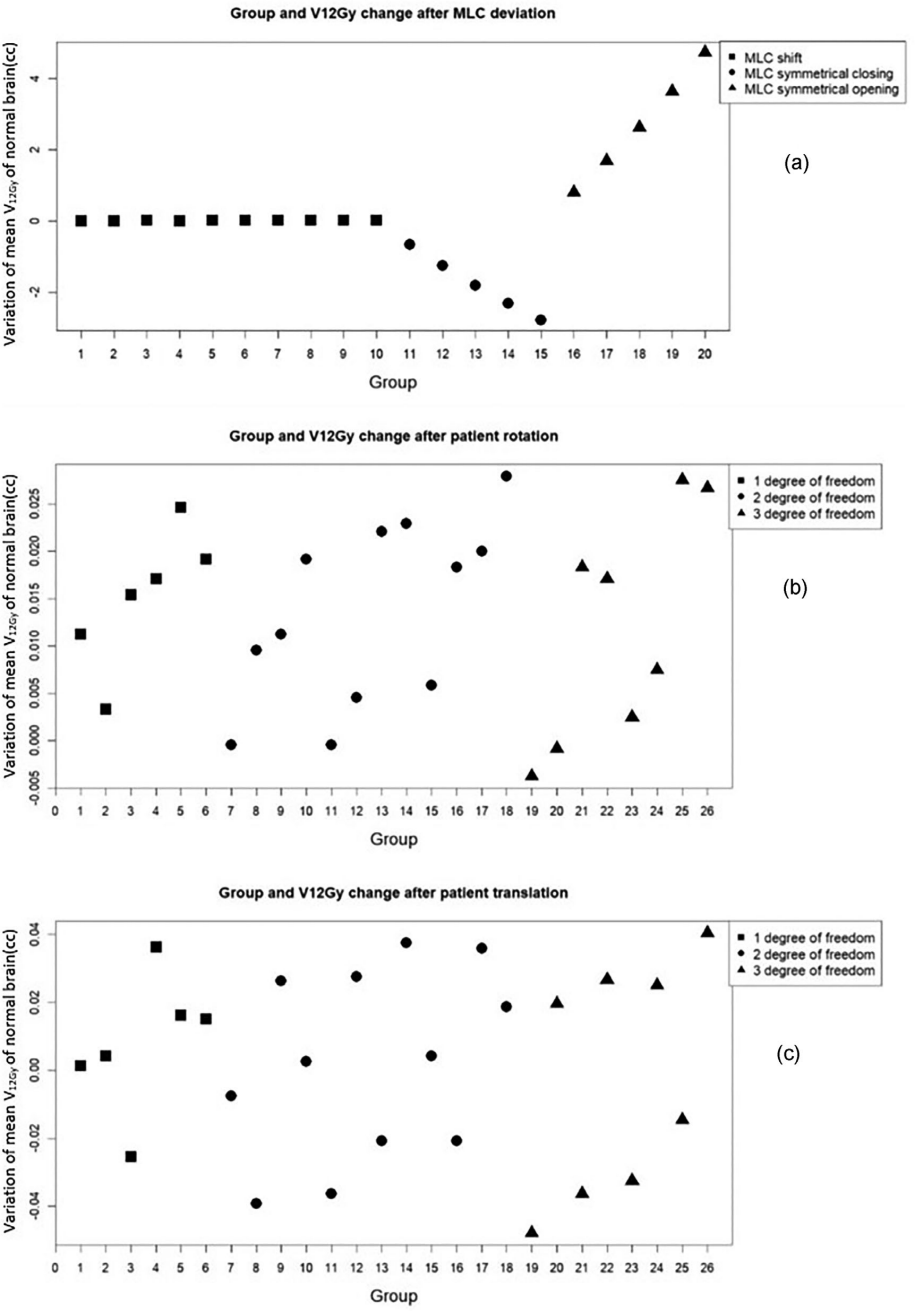
Variation in mean V_12Gy_ values of normal brain resulting from a) simulated MLC positional errors and b) simulated patient rotational errors and c) simulated patient translational errors. Mean V_12Gy_ does not change with MLC shift but is more sensitive to MLC symmetrical closing and opening. For rotational errors, V_12Gy_ only fluctuates slightly in a tiny range (from −0.005 to 0.0025). For translational errors, V_12Gy_ only fluctuates slightly in a small range (from −0.04 to 0.04). MLC groups 1–20 span C1 single‐sided shifts (Groups 1–10; left/right pairs at 0.2–1.0 mm), C2 symmetric closings (Groups 11–15), and C3 symmetric openings (Groups 16–20), each at 0.2–1.0 mm (see Table 4). Rotation groups (26 total) apply a 1° error in 1‐, 2‐, and 3‐DOF scenarios (6, 12, and 8 combinations); translation groups (26 total) apply a 1 mm offset in 1‐, 2‐, and 3‐DOF scenarios (6, 12, and 8 combinations). These are listed in Supplementary Tables A and B, respectively.

For normal brain tissue, the V_12Gy_ metric is primarily sensitive to MLC‐related errors. It increases slightly with MLC shifts and more substantially with symmetric MLC errors. The dosimetric change in V_12Gy_ follows a linear trend relative to MLC opening or closing: V_12Gy_ rises by about +5 cc per 1 mm with symmetrical MLC opening error, while decreases by 2.77cc/1 mm with symmetric closings (Figure 4a). In contrast, V_12Gy_ remains largely unaffected by patient setup uncertainties, with minimal changes under rotation (–0.005 to +0.0025 in Figure 4b) and translation (–0.04 to +0.04, Figure 4c). Overall, V_12Gy_ is considerably more influenced by symmetric MLC openings and closings than by shifts or patient setup uncertainties.

### 3.3 Margin selection

Understanding margin selection for plan robustness is crucial for HyperArc planning in the real world. In most cases, a minimum PTV margin of 1 mm in our simulation is sufficient to account for MLC positional deviations and patient setup uncertainties. However, certain patients may require larger margins to maintain adequate target coverage. In Table 4–12, we list average GTV D_100%_ coverage for all the cases (column “1 mm all”), worst cases (column “1 mm”), and the worst cases using larger margins (column 1.5 and 2 mm).

**TABLE 4:** Percentage of GTVs achieving D100% ≥ 100% after MLC errors, by error type, magnitude, and planning margin. “1 mm (all)” reports the GTV D100% coverage statistic across all lesions at the 1 mm PTV margin; “1 mm” reports the same statistic for the worst‐case lesion subset at the 1 mm PTV margin; “1.5 mm” and “2 mm” report the worst‐case subset with enlarged PTV margins.

			PTV margin			
MLC group No.	Error Type	Magnitude (mm)	1.0 mm (All)	1.0 mm	1.5 mm	2.0 mm
1–8	Shift (L/R)	0.2–0.8	99	93	93	100
9–10	Shift (L/R)	1.0	94–99	86	86–93	93
11	Sym. Closing	0.2	99	93	93	93
12	Sym. Closing	0.4	84	64	71	79
13	Sym. Closing	0.6	38	29	43	50
14	Sym. Closing	0.8	7	5.8	7.7	7.7
15	Sym. Closing	1.0	1.2	0	7.7	7.7
16–20	Sym. Opening	All	100	100	100	100

It is also essential to consider CI/GI when evaluating plan robustness and margin selection. For MLC positional errors, CI and GI showed increasing sensitivity as the magnitude of leaf error increased. For lateral leaf shifts, σ/μ increased from approximately 10%–11% at 0.2 mm, to 20%–24% at 0.4 mm, and up to 52%–72% at 0.6 mm, indicating substantial degradation in plan quality at larger MLC deviations. Systematic leaf shrinkage or expansion also produced large variations in CI and GI. For setup errors, the impact was strongly dependent on target size: for lesions < 0.5 cc, translational and rotational errors resulted in σ/μ values of approximately 43%–47% and 31%–36%, respectively, whereas for targets > 0.5 cc, the variability was much smaller (about 7%–10%). Overall, these additional analyses demonstrate that delivery and setup uncertainties can affect multiple clinically relevant SRS plan quality metrics, particularly for very small targets and larger MLC deviations.

Using 100% as a benchmark for adequate GTV D_100%_ coverage, a 2 mm margin is generally sufficient for most MLC shift errors (Table 4, Groups 1–10) but not for MLC closing errors, where coverage can fall well below 95% (Table 4, Groups 11–15). In contrast, MLC opening errors have no impact, making margins irrelevant for GTV coverage (Table 4, Groups 16–20). A 2 mm margin sometimes mitigates 1D and 2D patient rotational errors (Table S1, 1 and 2 DOF) but remains inadequate for 3D rotations (Table S1, 3 DOF). Similarly, a 2 mm margin is mostly sufficient for 1D patient translational errors (Table S2, 1 DOF), provides only partial protection for 2D translational errors (Table S2, 2 DOF), and performs poorly under 3D translational errors (Table S2, 3 DOF).

Notably, in worst‐case conditions, applying a 2.0 mm margin significantly enhances GTV robustness—particularly in mitigating the dosimetric impact of patient rotational and translational errors. These findings support the need for individualized margin assessment to ensure reliable target coverage under real‐world treatment uncertainties. On the other hand, increasing the margin from 1.0 mm to 2.0 mm results in a V_12Gy_ variation of normal brain tissue (Brain minus GTVs) of less than 1.0 cc in all cases. We also evaluated the dose to serial organs at risk (brainstem, optic chiasm, and optic nerves) as a function of margin expansion. Increasing the margin from 1 to 2 mm changed the dose to these structures by less than 0.3 Gy, with all values remaining within tolerance. Still, the dosimetric cost of larger margin on normal tissue may outweigh its benefit for rare extreme scenarios. This also supports that margin decisions should therefore be made through careful, case‐by‐case risk assessment.

## 4 DISCUSSION

Our study provides a comprehensive validation of the inherent robustness of SRS HyperArc treatments against a spectrum of uncertainties related to MLC as well as patient rotational and translational setup deviations. Our findings underscore the importance of considering factors such as lesion size and target distance to the isocenter to achieve desirable target coverage. After all, SRS plans are delivered in a single fraction. Unlike conventional radiotherapy, which benefits from the averaging effect over multiple fractions, SRS treatments do not have the opportunity to mitigate the impact of setup or delivery errors across sessions. This increases the importance of optimizing plan robustness from the outset.

Among all the simulated uncertainties, our data show that GTV D_100%_ is most sensitive to the symmetric MLC deviations (C2, C3), which result in the largest dosimetric impact (> 25%/1 mm). Then, the second most impactful factors are translational setup errors (C7–C9). Each additional translational DOF leads to ∼7.3% additional loss, indicating moderate‐to‐high sensitivity. GTV D_100%_ is least sensitive to rotational setup errors (C4–C6). Although a prior study^34^ suggests minimal correlation between DTI and dose coverage under MLC shifts or symmetric errors, our findings indicate that as patient translation and rotational deviations increase, DTI begins to exert a modest but measurable influence on dose distribution. Similarly, while volume does not significantly correlate with dose metrics under MLC shifts, larger target volumes can influence the slope of dose changes, particularly during MLC symmetric errors. Meanwhile, normal brain V_12Gy_ is primarily sensitive to symmetric MLC errors and largely insensitive to patient setup uncertainties, with changes considered less clinically significant than for GTV D_100%_. Plus, we also uncover the trivial interplay between the plan modulation factor and the robustness of HyperArc treatment.

The linear regressions provided in this work offer a practical reference for estimating the dosimetric impact of MLC and patient rotation uncertainties. Although quadratic regression models (D_100%_ = β_0_ +β_1_ ·Vol+β_2_ ·DTI^2^) were also evaluated, they provided no substantial difference in data interpolation. Therefore, we only include the linear models for their simplicity and ease of use.

We propose a clinically meaningful guideline for margin selection in HyperArc planning, emphasizing the need to align margin size with the magnitude of different types of baseline uncertainties. Our goal is to ensure that GTV coverage—specifically GTV D_100%_—remains at or above 100% of the prescribed dose across varying scenarios. While a 1.0 mm margin is commonly used in clinical practice for patients with multiple brain metastases, our simulation results show that plan robustness can often be compromised. Please note that the margin findings are scenario‐dependent simulation results derived from single error‐type perturbations applied in isolation, not compound clinical uncertainties; and a 2 mm margin does not adequately protect against worst‐case multi‐DOF translational and rotational errors. Clinicians should be mindful of this and increase the margin when necessary to maintain coverage.

Our results are broadly consistent with Fung et al's^35^ work for single or hypofractionated hyperArc plans, who assessed single‐fraction or hypofractionated HyperArc plans using 0.0, 1.0, and 2.0 mm PTV margins. In their study, a 1.0 mm margin sufficiently compensated for most interfractional translational and rotational movements, with minimal impact on normal brain tissue.

The robustness of single‐isocenter multi‐target intracranial SRS has been examined recently by May et al. for delivery errors^36^ and for patient setup errors^37^ using the TROG 2018 international planning‐challenge dataset, and by Prentou et al.^22^ ^.^ for MLC positional inaccuracy in Monaco‐planned single‐isocenter VMAT. The present study differs from this work in several respects relevant to clinical interpretation: it is restricted to clinical HyperArc plans with the HD‐120 MLC rather than a multi‐system challenge dataset; MLC errors are simulated by direct manipulation of leaf positions in the RT‐Plan DICOM file — a physical delivery error — rather than via a TPS modeling parameter; MLC delivery, rotational, and translational setup errors are evaluated within a single cohort and framework rather than in separate investigations; and the analysis of 123 lesions from 34 clinical plans is translated into an error‐type‐ and magnitude‐specific PTV margin assessment intended as a practical aid to plan review. The prior work cited above has already examined how plan complexity relates to robustness using established metrics. That was not a primary aim of this study. We therefore present our modulation analysis as a secondary, exploratory component. Our principal findings do not depend on it.

Beyond the mechanical and setup errors simulated in this study, intra‐fractional patient motion represents an additional source of dosimetric uncertainty in single‐isocenter SRS. Because all targets share a common isocenter, motion during beam delivery introduces displacement errors that scale with each target's distance from the isocenter. Yamada et al.^38^ quantified this effect in single‐isocenter multi‐target SRS, demonstrating that intra‐fractional displacement includes both distance‐dependent and distance‐independent components, and that biteplate immobilization substantially reduces displacement magnitude. Ohira et al.^25^ similarly showed that immobilization device selection meaningfully influences intra‐fractional motion. These findings complement our results: intra‐fractional motion introduces a time‐varying perturbation whose impact is greatest for peripheral targets. Future work incorporating dynamic motion modeling alongside the static error scenarios evaluated here would provide a more complete picture of delivery uncertainty in single‐isocenter SRS.

We acknowledge that a limitation of our work is the limited quantities used in the multiple linear regression modelling. The current regression is the average linear relationship across 123 lesions, with R^2^ and *p*‐values reported for GTV volume and DTI. However, individual lesion‐level variability — which is clinically important given the heterogeneity in target volume, count, and spatial distribution — is not explicitly characterized. The “worst case” column in Tables 4–6 provides some indication of outlier behavior but does not convey the distributional spread systematically.

Another limitation is the smaller number of plans created with 2 mm margins, which may reduce the statistical power for margin comparison. Moving forward, we aim to further explore this in future studies, for example, to develop a margin selection framework or lookup table based on lesion characteristics and expected uncertainties. Such a tool could aid clinicians in balancing treatment precision with robustness, supporting safer and more personalized SRS delivery.

## 5 CONCLUSION

Our data analysis shows that MLC motional uncertainty and patient positional uncertainty can lead to significant deterioration of the tumor target coverage in HyerArc SRS MBT plans. However, within the 1 mm positional uncertainty tolerance and 1 degree rotational uncertainty tolerance, there is no significant dosimetric variation for normal brain tissue. This work proposes a robustness analysis method for SRS MBT plans. It is imperative to analyze the robustness of each HyperArc SRS MBT plan prior to delivery to warrant reliable dose coverage for targets. Effects of target's size and distance to the plan isocenter on dosimetric variation were studied. Due to the weak correlation between target coverage variation and MLC positional uncertainty/patient positional uncertainty for different tumor sizes and distances to the plan iso‐center, a corresponding PTV with at least 1mm expansion from each individual GTV is strongly suggested for HyperArc MBT plans.

## AUTHOR CONTRIBUTIONS

Huijun Xu, Hong Zhang, and Baoshe Zhang conceived and designed the study, performed the data analysis, and drafted the original manuscript. All other authors contributed to data interpretation and critical revision of the manuscript. All authors reviewed and approved the final version of the manuscript.

## CONFLICT OF INTEREST STATEMENT

The authors have no conflicts of interest to disclose.

## ETHICS STATEMENT

This study was approved by the University of Maryland Institutional Review Board (no. HP‐00080058).

## Supporting information



Supporting Information
