# Supplementary material for: Evaluation of dosimetric impact and plan robustness of MLC motion and setup errors in SRS for multiple brain metastases
Source: J Appl Clin Med Phys. 2026 Jul 29;27(8):e70697. doi: 10.1002/acm2.70697 (PMC13419083; doi:10.1002/acm2.70697)
Supplement: Supplementary file 1 — Supporting Information [file ACM2-27-e70697-s001.docx]

**APPENDIX**

Supplementary Table S1. Percentage of GTVs achieving GTV D100% ≥ 100% after rotational setup errors, by DOF combination and planning margin.

| DOF | Rotation group No. | Yaw (°) | Pitch (°) | Roll (°) | No margin | 1.0 mm | 1.5 mm | 2.0 mm |
| --- | --- | --- | --- | --- | --- | --- | --- | --- |
| 1 DOF | 1 | −1 | 0 | 0 | 92 | 53 | 67 | 94 |
|  | 2 | 0 | −1 | 0 | 87 | 59 | 83 | 94 |
|  | 3 | 0 | 0 | −1 | 86 | 65 | 83 | 94 |
|  | 4 | 0 | 0 | 1 | 87 | 53 | 67 | 100 |
|  | 5 | 0 | 1 | 0 | 89 | 53 | 83 | 94 |
|  | 6 | 1 | 0 | 0 | 92 | 53 | 100 | 100 |
| 2 DOF | 7 | −1 | −1 | 0 | 72 | 47 | 67 | 94 |
|  | 8 | −1 | 0 | −1 | 53 | 29 | 50 | 88 |
|  | 9 | −1 | 0 | 1 | 62 | 24 | 75 | 94 |
|  | 10 | −1 | 1 | 0 | 65 | 47 | 58 | 94 |
|  | 11 | 0 | −1 | −1 | 64 | 29 | 67 | 94 |
|  | 12 | 0 | −1 | 1 | 61 | 35 | 83 | 94 |
|  | 13 | 0 | 1 | −1 | 55 | 35 | 67 | 94 |
|  | 14 | 0 | 1 | 1 | 61 | 24 | 67 | 71 |
|  | 15 | 1 | −1 | 0 | 72 | 41 | 50 | 94 |
|  | 16 | 1 | 0 | −1 | 56 | 29 | 75 | 94 |
|  | 17 | 1 | 0 | 1 | 60 | 35 | 50 | 100 |
|  | 18 | 1 | 1 | 0 | 66 | 47 | 67 | 94 |
| 3 DOF | 19 | −1 | −1 | −1 | 46 | 29 | 50 | 88 |
|  | 20 | −1 | −1 | 1 | 54 | 35 | 33 | 94 |
|  | 21 | −1 | 1 | −1 | 47 | 47 | 58 | 76 |
|  | 22 | −1 | 1 | 1 | 51 | 18 | 50 | 82 |
|  | 23 | 1 | −1 | −1 | 46 | 18 | 42 | 76 |
|  | 24 | 1 | −1 | 1 | 53 | 24 | 50 | 88 |
|  | 25 | 1 | 1 | −1 | 45 | 24 | 58 | 94 |
|  | 26 | 1 | 1 | 1 | 52 | 29 | 50 | 82 |

Supplementary Table S2. Percentage of GTVs achieving GTV D100% ≥ 100% after translational setup errors, by DOF combination and planning margin.

| DOF | Translation group No. | LR (mm) | AP (mm) | SI (mm) | No margin | 1.0 mm | 1.5 mm | 2.0 mm |
| --- | --- | --- | --- | --- | --- | --- | --- | --- |
| 1 DOF | 1 | −1 | 0 | 0 | 47 | 24 | 71 | 100 |
|  | 2 | 0 | −1 | 0 | 39 | 24 | 76 | 100 |
|  | 3 | 0 | 0 | −1 | 71 | 53 | 76 | 100 |
|  | 4 | 0 | 0 | 1 | 67 | 41 | 88 | 100 |
|  | 5 | 0 | 1 | 0 | 41 | 32 | 76 | 100 |
|  | 6 | 1 | 0 | 0 | 47 | 29 | 76 | 94 |
| 2 DOF | 7 | −1 | −1 | 0 | 1 | 0 | 18 | 71 |
|  | 8 | −1 | 0 | −1 | 2 | 0 | 29 | 100 |
|  | 9 | −1 | 0 | 1 | 4 | 0 | 29 | 82 |
|  | 10 | −1 | 1 | 0 | 3 | 2 | 35 | 100 |
|  | 11 | 0 | −1 | −1 | 6 | 6 | 53 | 76 |
|  | 12 | 0 | −1 | 1 | 0 | 0 | 41 | 100 |
|  | 13 | 0 | 1 | −1 | 4 | 1.8 | 29 | 76 |
|  | 14 | 0 | 1 | 1 | 1 | 0 | 35 | 100 |
|  | 15 | 1 | −1 | 0 | 6 | 2 | 47 | 88 |
|  | 16 | 1 | 0 | −1 | 5 | 0 | 41 | 82 |
|  | 17 | 1 | 0 | 1 | 6 | 0.06 | 0.29 | 0.88 |
|  | 18 | 1 | 1 | 0 | 2 | 0.12 | 0.47 | 0.76 |
| 3 DOF | 19 | −1 | −1 | −1 | 0 | 0 | 12 | 65 |
|  | 20 | −1 | −1 | 1 | 0 | 0 | 18 | 47 |
|  | 21 | −1 | 1 | −1 | 0 | 0 | 12 | 53 |
|  | 22 | −1 | 1 | 1 | 0 | 0 | 12 | 59 |
|  | 23 | 1 | −1 | −1 | 0 | 0 | 12 | 65 |
|  | 24 | 1 | −1 | 1 | 0 | 0 | 6 | 47 |
|  | 25 | 1 | 1 | −1 | 0 | 0 | 12 | 59 |
|  | 26 | 1 | 1 | 1 | 0 | 0 | 0 | 47 |
